# Strain discrimination of *Yersinia pestis* using a SERS-based electrochemically driven melting curve analysis of variable number tandem repeat sequences†
†Electronic supplementary information (ESI) available: Electrochemical melting curves along with 95% confidence intervals for the three PCR amplicons hybridized to probe-1, fluorescence melting curves for amplicons hybridized to probe-2 in the presence of a blocker oligonucleotide, structure of the thiol linker. See DOI: 10.1039/c4sc03084b


**DOI:** 10.1039/c4sc03084b

**Published:** 2015-01-07

**Authors:** E. Papadopoulou, N. Gale, S. A. Goodchild, D. W. Cleary, S. A. Weller, T. Brown, P. N. Bartlett

**Affiliations:** a Chemistry , University of Southampton , Highfield , Southampton SO17 1BJ , UK . Email: pnb@soton.ac.uk; b ATDBio Ltd , Chemistry , University of Southampton , Highfield , Southampton SO17 1BJ , UK; c DSTL , Wiltshire SP4 0JQ , Salisbury , Porton Down , UK; d Department of Chemistry , University of Oxford , Chemistry Research Laboratory , Oxford OX1 3TA , UK

## Abstract

Strain discrimination within genetically highly similar bacteria is critical for epidemiological studies and forensic applications. An electrochemically driven melting curve analysis monitored by SERS has been utilised to reliably discriminate strains of the bacterial pathogen *Yersinia pestis*, the causative agent of plague. DNA amplicons containing Variable Number Tandem Repeats (VNTRs) were generated from three strains of *Y. pestis*: CO92, Harbin 35 and Kim. These amplicons contained a 10 base pair VNTR repeated 6, 5, and 4 times in CO92, Harbin 35 and Kim respectively. The assay also included a blocker oligonucleotide comprising 3 repeats of the 10-mer VNTR sequence. The use of the blocker reduced the effective length of the target sequence available to bind to the surface bound probe and significantly improved the sensitivity of the discrimination. The results were consistent during three replicates that were carried out on different days, using different batches of PCR product and different SERS sphere segment void (SSV) substrate. This methodology which combines low cost, speed and sensitivity is a promising alternative to the time consuming current electrophoretic methods.

## Abstract 2

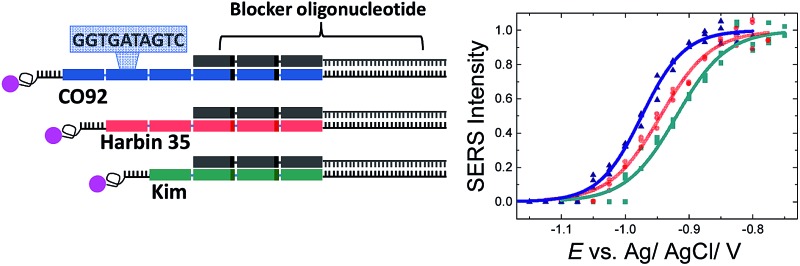
Variable number tandem repeats in DNA extracted from three bacterial *Y. pestis* strains have been differentiated using *E*-melting analysis monitored by SERS, combined with the use of a blocker oligonucleotide.

## Introduction

Strain identification within bacterial organisms is essential in forensic applications of pathogen outbreaks as well as to ensure prevention and control of infections.^1^–^3^ However, a number of pathogenic bacteria are genetically highly similar, making strain discrimination difficult. A number of methods targeting small genetic variations between bacterial species have been defined to perform the highest resolution possible such as Variable Number Tandem Repeats (VNTRs) typing, Different Regions (DFRs), Insertion Sequence (IS), Clustered Regularly Interspaced Short Palindromic Repeats (CRISPRs) and Single Nucleotide Polymorphisms (SNPs).^1^,^4^–^8^ VNTRs, also known as minisatellites, are short oligonucleotide sequences in the range of 6–100 base pairs (bp) that are repeated multiple times within a genome. The repetitive nucleotide sequences at the same locus can be identical or differ slightly. The polymorphic nature of VNTR regions in bacterial genomes allows them to be used as markers to differentiate strains within genetically very similar bacteria as well as to provide epidemiological and phylogenetic information.^4^,^9^–^12^

The most widely used methods for VNTR analysis are based on electrophoretic separation that measures the size in different alleles such as Multiple Locus Variable number repeat Analysis (MLVA) which involves PCR amplification of the selected VNTR loci followed by capillary electrophoresis.^13^–^16^ This approach, despite being highly discriminating, is time consuming and requires specialised equipment and skilled technicians. Simpler methods utilise melting curve analysis, which discriminates the DNA fragments on the basis of the differences in their melting temperature. HyBeacons®, which are fluorescent oligonucleotide probes that emit greater amounts of fluorescence after hybridization, have been used for a fluorescence-based differentiation and identification of short tandem repeats (repeat units 3–9 bp).^17^,^18^

We have developed an electrochemically induced DNA denaturation approach that is monitored by SERS. Similar to fluorescence-based melting curve analysis, we can construct potential melting curves by measuring the SERS intensity as a function of applied potential. In this approach, the DNA target is labelled with a Raman fluorophore and then hybridized to a DNA probe that is immobilised on an ordered sphere segment void (SSV) gold surface *via* three dithiol linkers. It has been previously shown that SSV surfaces, which are made by electrodeposition of gold around closely packed monolayers of spheres, can provide large and reproducible SERS enhancements.^19^–^22^ Upon application of an increasing negative potential on the SSV surface, the dsDNA denatures and the denaturation can be monitored by following the decrease in intensity of the Raman signal from the labelled target DNA as it diffuses away from the surface. This SERS-based methodology has been successfully used for the discrimination of mutations in the cystic fibrosis *trans*-membrane regulator gene^23^,^24^ and short tandem repeats in the D16S539 locus of the human genome.^25^ In those cases the target DNA was amplified using synthetic templates.

We have recently extended the application of this technique for exploitation of molecular assays for discriminating between DNA from closely related microorganisms.^30^ The target organism chosen for study was *Yersinia pestis*, which is the aetiological agent of the systemic, invasive disease known as plague; an ancient disease of substantial historic importance resulting in the deaths of millions worldwide.^26^–^29^ *Y. pestis* also remains of interest to the defence and security community due to the potential for use as a biological weapon. The rapid discrimination of different species is therefore important for epidemiological studies and for forensic applications of importance to national security.^2^ This organism represents a particular challenge for the generation of simple molecular assays as it shares a high degree of similarity to other *Yersinia* spp. Therefore assays which discriminate within the *Yersinia* genus such as Single Nucleotide Polymorphism (SNP) or VNTR provide highly resolved lineage information for both evolutionary and epidemiological analysis.

**Fig. 1:**
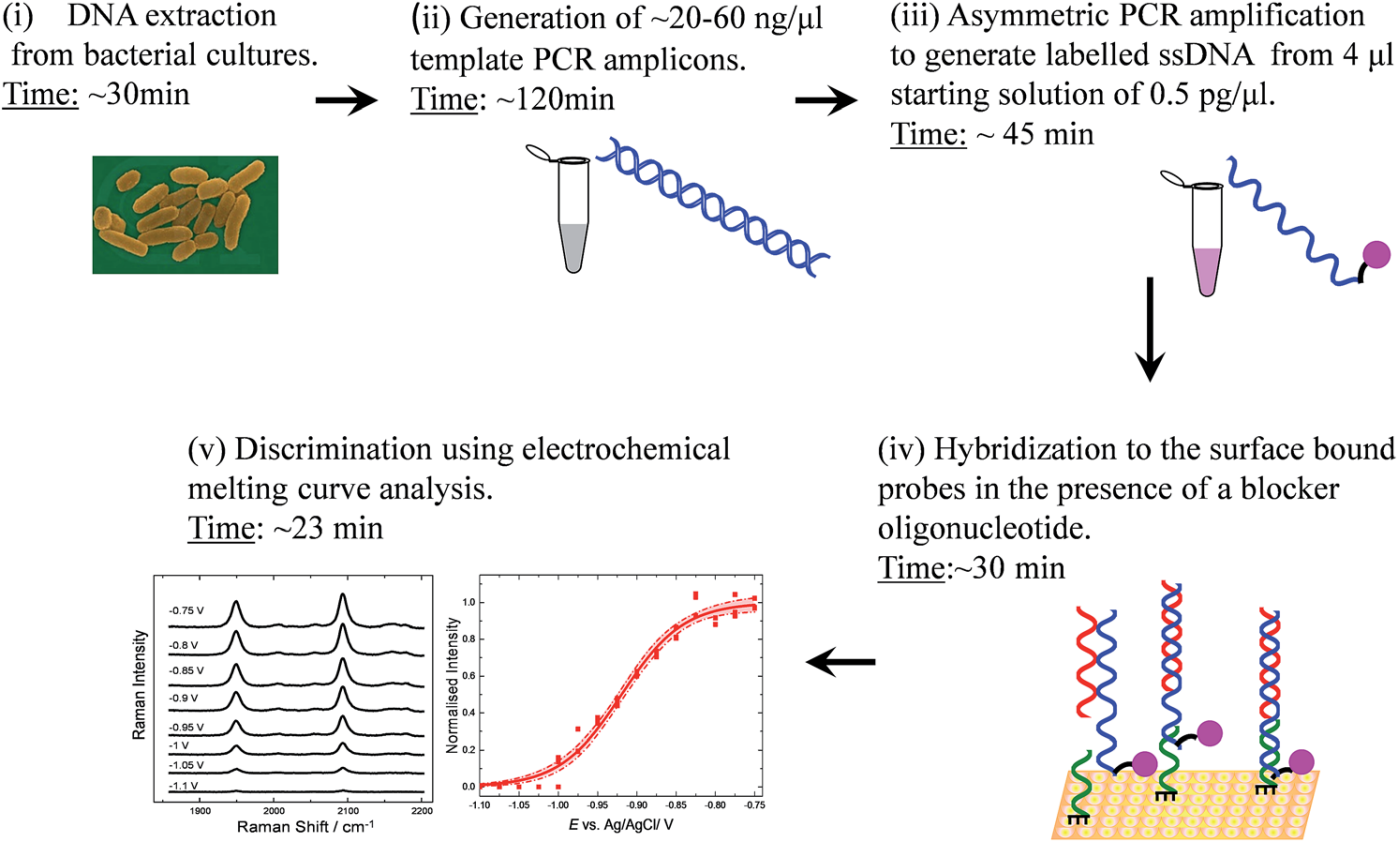
Schematic flow diagram of the SERS detection assay illustrating each operational step involved. First, the DNA is extracted from bacterial cultures and the amplified DNA product is used as a template to generate single stranded labelled DNA during asymmetric PCR. The step (ii) could be omitted for future assays as it is possible to perform asymmetric PCR on genomic DNA directly.^31^ The labelled target is then hybridized to surface bound probes in the presence of a blocker oligonucleotide. Following electrochemical analysis monitored by SERS the pathogenic strains can be discriminated on the basis of the differences in their melting potential.

In a previous study we targeted discrimination of *Y. pestis* from the closely related *Y. pseudotuberculosis*. This was achieved on the basis of species specific SNPs present within long (>250 bp) DNA PCR amplicons.^30^ In this study, we further exemplify the strength of this technique in molecular typing of bacterial species to achieve strain differentiation for *Y. pestis*. We show the differentiation of three strains of *Y. pestis* by VNTR analysis based on different numbers of repeats of a VNTR sequence present within these strains. Their VNTRs can be differentiated on the basis of the differences in their melting potential. Similarly to our previous assays, the PCR amplicons were used unpurified. An outline of the strategy is illustrated in Fig. 1. Specifically, genomic DNA was extracted and amplified from the polymorphic bacterial strains CO92, Harbin 35 and Kim that had a 10 bp repeat unit, repeated 6, 5 and 4 times respectively, for each strain. To improve the discrimination efficiency of the three bacterial strains a blocker oligonucleotide was employed to reduce the effective length of the target sequence available to bind to the surface bound probe. Hence, an increase in the differences in melting potential between the amplicons was achieved. To our knowledge this is the only SERS-based sensor that can be applied in VNTR genotyping.

## Experimental

### Identification of VNTRs and extraction of DNA

Primers for the identification of VTNR loci were selected from Le Fleche *et al.*^9^ Genomic DNA from *Y. pestis* strains CO92, Harbin 35 and Kim was extracted from overnight cultures of each strain using a QIAamp DNA mini-prep kit (Qiagen, UK) according to the manufacturer's instructions.

### Generation of template PCR amplicons

PCR was performed in 25 μl reaction volumes on a GeneAmp® PCR System 9700 (Applied Biosystems, UK). Reactions contained: 0.2 μM of forward and reverse primer, 1× PCR buffer without Mg^2+^, 3 mM MgCl_2_ (Sigma-Aldrich, UK), 80 mM each dNTP (Roche, UK), 0.04 U μl^–1^ Taq polymerase (Roche) and 18.4 μl molecular grade dH_2_O (Sigma-Aldrich, UK). PCR conditions were 94 °C for 5 min; 35 cycles of 95 °C for 30 s, 58 °C for 30 s and 72 °C for 30 s; 72 °C 7 min. E-gels® (Invitrogen, Life Technologies, UK) were used to visualise amplicons. E-gels® were used according to the manufacturer's instructions. Visualisation was performed using a Chemi HR410 BioSpectrum® Imaging System (Ultraviolet Products Ltd, UK).

### Asymmetric PCR amplification to generate DNA amplicons labelled with Texas Red

Template DNA was further amplified to label the template with Texas Red using asymmetric PCR conditions. The PCR reactions were performed using a thermocycler (Eppendorf Mastercycler Gradient) and they were carried out in 20 μl volumes each containing 10 μl of 2× BioRad SsoFast™ Probe Supermix, 0.5 μM Texas Red labelled forward primer, 0.05 μM reversed primer and 0.5 pg μl^–1^ of the amplicon. The amplification was monitored by adding 0.6 μl of Sybr green (Biorad) to the reaction volume. PCR conditions were 95 °C for 2 min, followed by 40 cycles of 95 °C for 1 s and 55 °C for 1 s.

### Oligonucleotide synthesis

Oligonucleotides were synthesised using standard methods by ATDBio Ltd (Southampton, United Kingdom). This included the PCR primers (the forward primer was labelled with Texas Red at the 5′ end *via* aminohexyl linker), the blocker and DNA probes (Table 2). The probes had a dithiol anchor modification at the 3′ end (Fig. S1†) using dithiol phosphoramidite (DTPA, Glen research, Virginia, USA). This linker has 3 dithiol groups and is chosen to ensure bonding of the probe to the gold at the high negative potentials used.

### Preparation of sphere segment void (SSV) substrates

A monolayer template of 600 nm polystyrene spheres (Fisher Scientific as a 1% wt aqueous suspension) was formed at the surface of a gold coated microscope slide using a convective assembly method.^23^ Gold was deposited through the template to a height of 480 nm at –0.72 V *vs.* SCE from commercial gold plating solution (ECF 60, Metalor) containing 100 μl brightener (E3, Metalor) in 20 ml of plating solution. After deposition the polystyrene spheres were removed by immersion in DMF (HPLC grade, Rathburn Chemicals Ltd, Scotland) for thirty minutes, and the substrates were rinsed in deionised water before immediate use.

### Electrochemically driven melting experiments

The DNA probes were immobilised on the SSV substrates by immersing the substrates in DNA solutions (1 μM in 10 mM Tris/1 M NaCl) overnight. Following thorough rinsing, the remaining surface was passivated by immersing the substrates in a solution of mercaptohexanol (10 mM in 10 mM Tris/1 M NaCl). To hybridize the immobilized DNA probes, the substrates were heated in the PCR solutions to 90 °C for 10 min and then were allowed to cool slowly to room temperature in a large water bath. In addition to the labelled ssDNA amplicon, the PCR buffer also contained the TAG polymerase, salts, dNTPs, DNA primers and amplicon dsDNA sequences. After hybridization, the substrates were rinsed thoroughly and stored in the buffer until further analysis. For the second assay, 0.45 μM of the blocker oligonucleotide was added in the PCR solution. Electrochemical melting experiments were carried out in a custom-built spectro-electrochemical Raman cell (Ventacon Ltd.) where the SSV substrate is used as the working electrode, a platinum wire as the counter electrode, and Ag/AgCl pellet as the reference electrode. In a typical electrochemical melting experiment, the potential was swept at 0.7 mV s^–1^ from a starting potential of –0.4 to –1.2 V in 10 mM Tris/1 M NaCl buffer. All electrochemical measurements were carried out using an EcoChemie AutolabIII potentiostat/galvanostat at room temperature. Raman spectra were acquired using a Renishaw 2000 microscope instrument equipped with a 632.8 nm He–Ne laser. Typically, the laser power was 2.3 mW and spectra were recorded with an exposure time of 30 s.

### Data analysis

SERS spectra presented were baseline-corrected using a polynomial multipoint fitting function in Origin 9.1. The Raman intensities of the peaks are taken as height above the baseline. Origin 9.1 was used to fit sigmoidal curves to the melting profiles using the following expression1
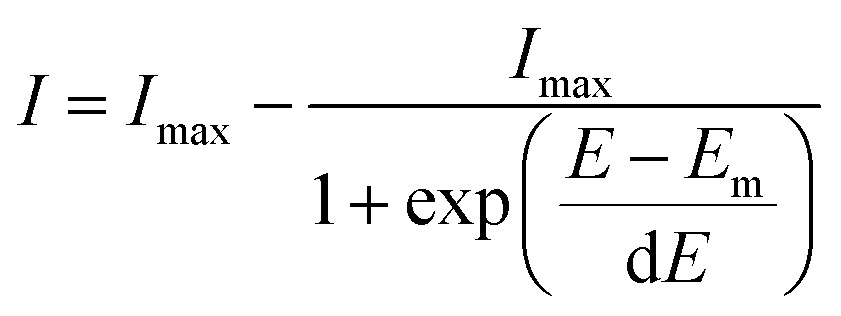
where *I* is the absolute spectral intensity of the band at 1505 cm^–1^ at the applied potential *E*, *I*_max_ is the average intensity value at the plateau for the sigmoidal curve, *E*_m_ is the melting potential when *I* equals *I*_max_/2, and d*E* is a constant that describes the sharpness of the melting curve (the gradient of the curve at *E*_m_ is *I*_max_/4d*E*).

## Results and discussion

The *Y. pestis* species has historically been divided into three key subgroups termed biovars based on the ability to ferment glycerol and reduce nitrate. The human pathogenic biovars; Antiqua, Medievalis and Orientalis are associated with the various branches of *Y. pestis* phylogeny and represent divergence from an ancestral strain.^32^ For this study, genomic DNA was extracted from *Y. pestis* strains covering these biovars namely Kim and Harbin35 (Medievalis), CO92 (Orientalis) and Nepal (Antiqua). Primary PCR products were amplified from the genomic DNA that contained a 10 nucleotide VNTR. The VNTR sequence was repeated 6 times for CO92, 5 times for Harbin35 and Nepal and 4 times for Kim. The second and third 10 nucleotide repeat units of the sequences starting from the 5′ end of each DNA amplicon (Table 1) differed from the rest of the repeat units by one base, *i.e.* the first base in the repeat unit was A instead of G. As the VNTR was identical for Harbin 35 and Nepal, the DNA amplicon for Harbin 35 was taken forwards to cover both strains. Due to the shared sequence it can be assumed that the outputs of the assay would be identical for both strains.

**Table 1:** Oligonucleotide sequences (5′–3′) showing the PCR fragments for the three bacterial strains, probe 1 and 2 and blocker oligonucleotide combined with probe 2. Forward primer sequences are shown on the amplicons in bold and reverse primers were the reverse complements of the sequences in bold and underlined, for both assays. The positions where the repeat units differ in terms of additional nucleotide polymorphisms are underlined. H = hexaethylene glycol, X = dithiol

Assay combined with probe-1	
CO92	CCTATACCGCTACGATCAGCCTCTATCGCCAAT**CACTATCATCAACCATCAAC**-GACTATCACC-AACTATCACC-AACTATCACC-GACTATCACC-GACTATCACC-GACTATCACC-**AACTACCTGCGACTGGTAG**-CCCCAAATATCA
Harbin35	CCGTTACCGCTACGATCAGCCTCTATCGCCAAT**CACTATCATCAACCATCAAC**-GACTATCACC-AACTATCACC-AACTATCACC-GACTATCACC-GACTATCACC-**AACTACCTGCGACTGGTAG**CCA
Kim	CTCGTTACCGCTACGATCAGCCTCTATCGCCAAT**CACTATCATCAACCATCAAC-**GACTATCACC-AACTATCACC-AACTATCACC-GACTATCACC-**AACTACCTGCGACTGGTAG**CCCCAAATATCAGA
Probe-1	GGTGATAGTC-GGTGATAGTC-GGTGATAGTC-GGTGATAGTT-GGTGATAGTT-GGTGATAGTC-HXXX
Assay combined with probe-2	
CO92	TGATATTTGG**GGCTACCAGTCGCAG**GTAGTT-GGTGATAGTC-GGTGATAGTC-GGTGATAGTC-GGTGATAGTT-GGTGATAGTT-GGTGATAGTC-GTTGATGGTT**GATGATAGTGATTGGCGATA**GAGGCTGATCGTAGCGGTATAGG
Harbin35	T**GGCTACCAGTCGCAG**GTAGTT-GGTGATAGTC-GGTGATAGTC-GGTGATAGTT-GGTGATAGTT-GGTGATAGTC-GTTGATGGTT**GATGATAGTGATTGGCGATA**GAGGCTGATCGTAGCGGTAACGG
Kim	TCTGATATTTGG**GGCTACCAGTCGCAG**GTAGTT-GGTGATAGTC-GGTGATAGTT-GGTGATAGTT-GGTGATAGTC-GTTGATGGTT**GATGATAGTGATTGGCGATA**GAGGCTGATCGTAGCGGTAACGAG
Probe-2	GACTATCACC-GACTATCACC-GACTATCACC-AACTAC-HXXX
Blocker	TATCGCCAATCACTATCATCAACCATC-AACGACTATCACC-AACTATCACC-AACTATCACC

Amplified DNA was used as a template for asymmetric PCR to generate ssDNA amplicons carrying a SERS active reporter chromophore (Texas Red) at the 5′ end. It is worth mentioning that asymmetric PCR can be performed on genomic DNA directly for future assays,^31^ however primary template amplicon is produced here for health and safety reasons. The labelled target DNA was hybridized to the surface bound DNA probe directly from the PCR buffer.

**Fig. 2:**
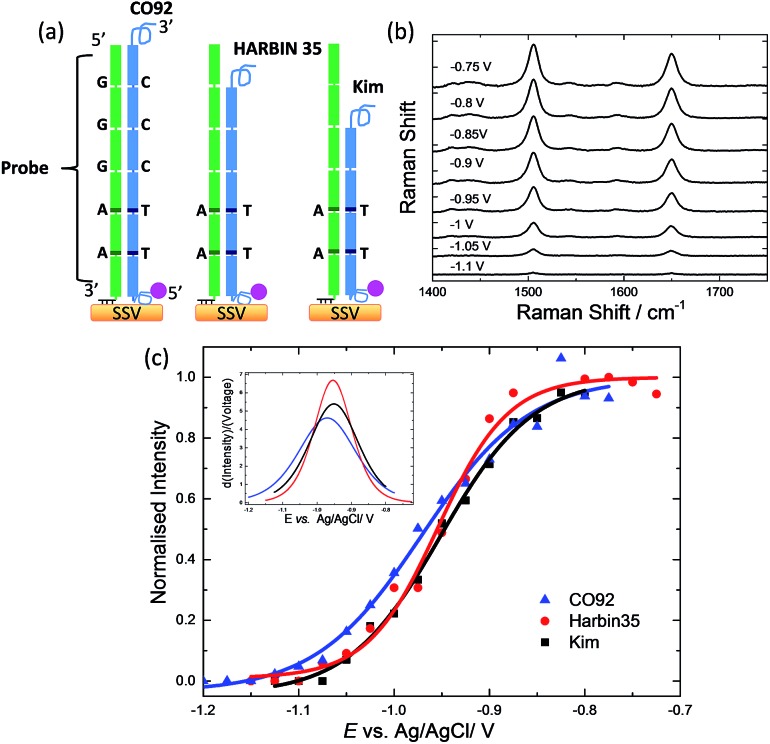
(a) Schematic representation of the PCR amplicons hybridised to the immobilised on the SSV substrate probe-1. The darker lines illustrate the position of the one base difference (a G-C bp is replaced by an A-T bp) in the two 10 bp repeat units compared to the rest of the units. (b) SERS of Texas Red for the PCR fragment from CO92 bacterial strain hybridised to probe-1. The spectra have been reported at different applied potentials *vs.* Ag/AgCl as shown in the figure. Spectra were acquired with 633 nm excitation laser and have been background subtracted and normalised with respect to the laser power and accumulation time. (c) *E*-melting curves from CO92 (6 repeat units), Harbin 35 (5 repeat units), and Kim (4 repeat units) hybridized to probe-1. Each set of data was fitted to eqn (1). The potential was swept at a scan rate of 0.7 mV s^–1^ in 10 mM Tris buffer (pH 7.2) containing 1 M NaCl.

The initial hybridization probe (probe-1) was designed to comprise of 6 repeat units (Fig. 2(a)), where two of the units differ by one base (T instead of C) in order to be perfectly complementary to the targets. It was assumed that the difference of two nucleotides within the repeat units would be adequate to ensure specific hybridization of the PCR amplicons to the probes and prevent probe slippage along the tandem repeat targets. The probe was immobilised on the SSV Au surfaces *via* three dithiol linkers on the 3′ end to ensure that it is strongly bound to the surface. The surface was then passivated by treatment with mercaptohexanol to prevent the non-specific adsorption of DNA and allow the DNA probes to adopt perpendicular orientation on the surface.^33^ Target DNA labelled with Texas Red was hybridized with the immobilised probe. In this orientation the fluorophore would be situated close to the surface while having small overhanging sequences (∼20 bases) at both ends. A schematic of the design is shown in Fig. 2(a). Hybridization of probe-1 to the PCR amplicons yields a 60, 50 and 40 bp duplex for the CO92, Harbin 35 and Kim strain respectively.

Good signal to noise spectra of the Texas Red labelled DNA was recorded after hybridization of the surface bound probes. Application of cathodic potentials from –0.4 V to –1.4 V *vs.* Ag/AgCl changed the intensity of the Texas Red Raman bands that initially increased and then decreased sharply (see ESI, Fig. S2†). The initial increase in intensity is reversible and is most likely due to a change in orientation of the label with respect to the SSV surface.^25^ The subsequent decrease in intensity beyond –0.7 V is due to the electrochemically induced denaturation of the DNA duplexes that allows the labelled DNA target to diffuse away from the surface (Fig. 2(b)) The raw spectra, without baseline correction, are shown in the ESI, Fig. S3.† The intensity of the most intense Texas Red band (1505 cm^–1^) was plotted against the applied potential for each PCR amplicon and the data were fitted to eqn (1) in order to define the melting potential (*E*_m_) (Fig. 2(c)). The *E*_m_ value is the potential at which the intensity of the band at 1505 cm^–1^ is half the intensity value at the plateau of the sigmoidal curve. In these experiments the difference between the *E*_m_ values was less than 10 mV, which is inadequate to secure reliable discrimination of the three bacterial strains. In addition, taking into account the 95% confidence intervals (Fig. S4,† the width of the confidence intervals is proportional to the standard error of the predicted absolute signal intensity (*I*) value), it is evident that the discrimination is not reliable.

It was considered likely that the weak discrimination observed using this probe design was due to a poorly controlled binding of the VNTR sequences along the immobilised probe. It is possible that the difference of two single nucleotides within the VNTR repeat units was not adequate to stabilise the position of the duplex and prevent probe slippage. The use of a short additional anchor sequence was thus considered. However, the use of an anchor sequence with the current design would increase the length of the duplexes, thus decreasing the difference in melting potential (Δ*E*_m_) between the three PCR fragments and rendering the discrimination more difficult.

Instead, a new design using a combination of both anchor sequence and a blocker oligonucleotide was employed to reduce the effective length of the target sequence available to hybridize to the surface bound probe. Thus, the use of a shorter probe was allowed onto which an anchor sequence was added to secure precise hybridization of the probe to the PCR amplicons. The use of a blocker oligonucleotide has been evaluated in assays utilising HyBeacon® probes (fluorescence probes) and fluorescence melting analysis. It has been shown to permit the reliable differentiation of high numbers of repeats for forensic loci by effectively increasing the difference in melting temperature (*T*_m_) between STRs of similar length.^17^,^34^

### *E*-melting analysis with the use of a blocker

**Fig. 3:**
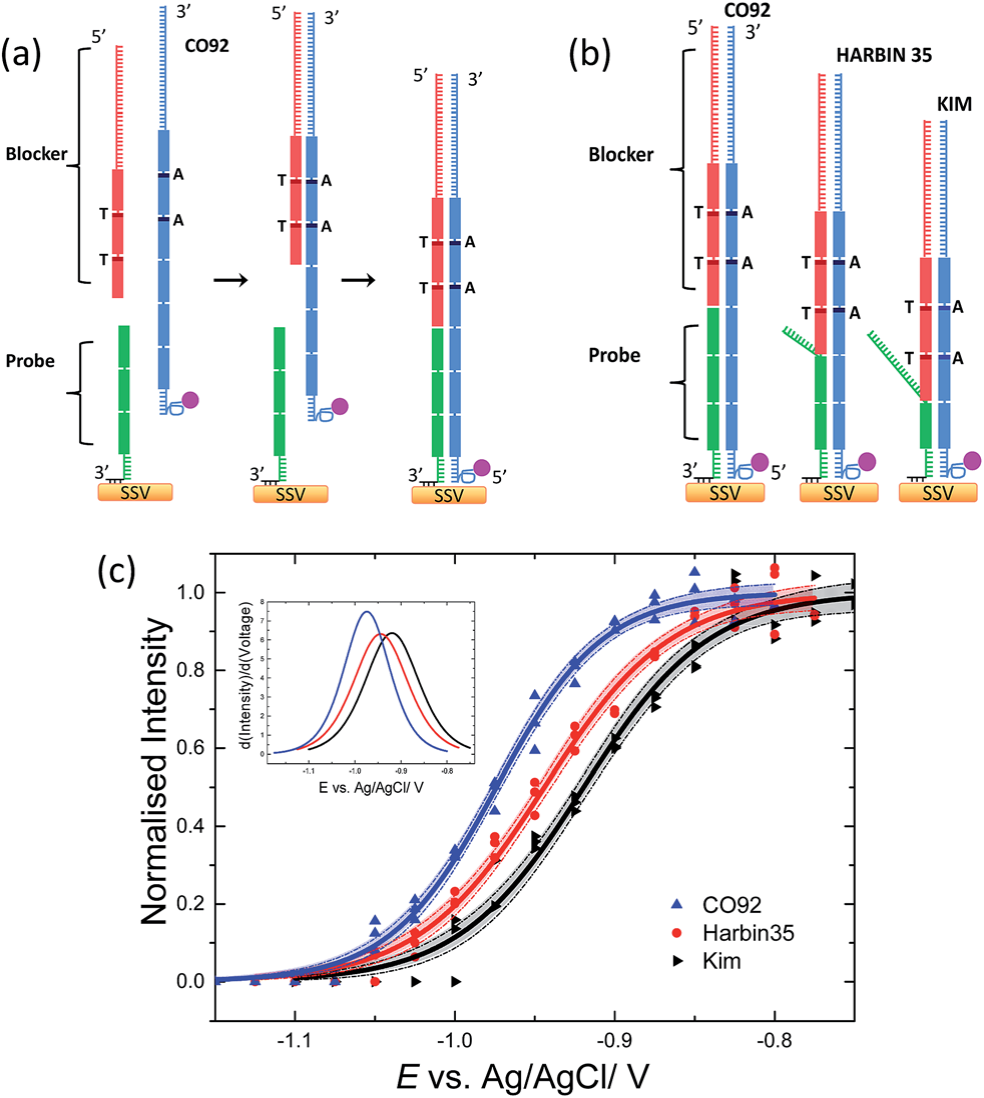
(a) Schematic representation illustrating the hybridization order for CO92 (6 repeat units), to probe-2 in the presence of a blocker oligonucleotide. The 3′ end of the blocker oligonucleotide (red) is composed of three repeat units, two of them have one base difference compared to the third (illustrated by the use of darker lines), followed by a 30 bases anchor at the 5′ end. The 3′ end of the probe (green) is modified with three dithiol and a hexaethylene glycol linker, and is composed of a 6 bases anchor followed by three repeat units. The blocker oligonucleotide (red) is hybridized to the DNA target prior to the surface immobilised probe-2. (b) Binding of the CO92, Harbin 35 and Kim amplicons to probe-2. (c) Solid lines represent the *E*-melting curves and dotted lines the 95% confidence limits, from CO92 (6 repeat units), Harbin 35 (5 repeat units), Kim (4 repeat units) hybridized to probe-2. The potential was swept at a scan rate of 0.7 mV s^–1^ in 10 mM Tris buffer (pH 7.2) containing 1 M NaCl.

The new probe (probe-2) was designed to comprise 3 repeat units and a 6 bases anchor at the 3′ end which was modified with three dithiol linkers. The ssDNA amplicon for hybridization was the reverse complement to that used in the previous design, thus the second and third repeat units starting from the 3′ end of the amplicon (instead of starting from the 5′ end as previously) differed from the rest of the units by one base. This design allowed the identical repeat units to be positioned closer to the surface, allowing the sequences with one base difference to hybridize to the blocker oligonucleotide. The blocker oligonucleotide comprised 3 repeat units (2 of them had one base different) and a 30 bases non-repetitive sequence that functioned as an anchor to prevent slippage of the blocker unit. Fig. 3(b) demonstrates the experimental design. Fluorescence melting studies demonstrated that the *T*_m_ of the blocker oligonucleotide was higher than that of the probe (Fig. S5†). More specifically the *T*_m_ of the blocker oligonucleotide was ∼74 °C while the *T*_m_ of the full length probe-2 bound to the longest amplicon (CO92) was 70 °C. This ensured that the blocker oligonucleotide would have hybridized to the DNA amplicons prior to the probe permitting only 3, 2 and 1 repeat units from the 6, 5 and 4 repeat units respectively to hybridize to the surface bound probe for each strain. The order of hybridization is illustrated in Fig. 3(a) for the DNA target with 6 repeat units (CO92).

Three sets of data were collected for the electrochemically driven melting of each amplicon. Each data set was collected on different days, using different batches of PCR products and SSV substrates. The three sets of data for each amplicon were analysed as described previously^30^ in order to produce a single common melting curve for each set of replicate experiments together with the corresponding 95% confidence interval. Briefly, each individual data set was normalised by its *I*_max_ value, which is the intensity value at the plateau of the sigmoidal curve. The normalised sets of data for each amplicon were fitted to eqn (1) using a weighted global fit, where each data set was weighted in inverse proportion of its particular value of *I*_max_^2^. The *E*-melting curves, for the three amplicons, along with the 95% confidence intervals are shown in Fig. 3(c). It is clear; taking into account the confidence intervals, that reliable discrimination of the three bacterial strains has been achieved. The use of a blocker oligonucleotide resulted in an increased difference in the *E*_m_ between the amplicons (Table 2) that improved the sensitivity of the assay significantly.

**Table 2:** Melting potentials (*vs*. Ag/AgCl) and d*E* values for CO92 (6 repeat units), Harbin 35 (5 repeat units), Kim (4 repeat units) hybridised to probe-2 in the presence of a blocker oligonucleotide, determined from the global fitting of the three data set collected for each amplicon to eqn (1)

	CO92	Harbin 35	Kim
*E*_m_/V	–0.974 ± 0.002	–0.944 ± 0.003	–0.919 ± 0.003
d*E*/V	0.039 ± 0.003	0.040 ± 0.002	0.033 ± 0.002

Utilising a SERS-based electrochemically driven melting analysis for the discrimination of tandem repeats is a promising alternative over the conventional methods that are based on electrophoretic separation that measures the size in different alleles. This methodology does not require expensive equipment and has the potential to be incorporated into portable technology. More importantly, SERS has the capability to provide high degree of multiplexing due to the sharper vibrational bands provided from the Raman labels which can be synthetically attached to the DNA.^35^,^36^ In addition the SERS enhancement is distance dependent, therefore only the SERS signal for the label attached to the DNA when is in close proximity to the surface can be detected. This is an advantage over fluorescence melting since we can perform the discrimination using the PCR buffer and the only melting peaks detected originate from the probe melting, which simplifies sample analysis. In more detail, the blocker oligonucleotide is bound to an unlabelled part of the amplicon, therefore its melting peak is not detected by SERS. In addition the labelled, full length duplex amplicons that exist in the PCR solution cannot be adsorbed on the surface due to passivation of the surface with mercaptohexanol after immobilisation of the thiolated probes, therefore their melting curves are not detected. Finally the method is highly sensitive, here we show discrimination of VNTRs from 4 μl starting solutions of 55–65 pM for each amplicon in a total time of ∼100 min for asymmetric PCR amplification, hybridization to the surface bound probe and electrochemically driven melting.

## Conclusions

VNTRs in DNA extracted from three bacterial *Y. pestis* strains (CO92, Harbin 35 and Kim) have been differentiated using *E*-melting analysis monitored by SERS, combined with the use of a blocker oligonucleotide. Genomic DNA was extracted from the three bacterial strains and then amplified and labelled using conventional PCR. The PCR products were used to hybridize surface bound probes with no further purification. A blocker oligonucleotide was hybridized to the target sequences to reduce the number of repeat units available to hybridize to the surface bound probe. The use of the blocker permits clear and reliable differentiation of VNTRs. The results were consistent during three replicates that were carried out on different days, using different batches of PCR product and different SSV substrate. To the best of our knowledge this is the only SERS-based methodology that has been used to discriminate bacterial DNA sequences *via* tandem repeat analysis. We believe that this technology is a promising alternative to current electrophoretic methods, and could also have utility in the development of a portable device used to rapidly discriminate strains within highly related homogenous bacterial species.

## Supplementary Material

Supplementary information
