# Supplementary material for: Strain discrimination of Yersinia pestis using a SERS-based electrochemically driven melting curve analysis of variable number tandem repeat sequences
Source: Chem Sci. 2015 Jan 7;6(3):1846–52. doi: 10.1039/c4sc03084b (PMC5701729; doi:10.1039/c4sc03084b)
Supplement: Supplementary file 1 [file SC-006-C4SC03084B-s001.pdf]

Electronic Supplementary Material (ESI) for Chemical Science.
This journal is © The Royal Society of Chemistry 2015

Strain discrimination of *Yersinia pestis* using a SERS-based electrochemically driven melting curve analysis of variable number tandem repeat sequences

E. Papadopoulou,[a] N. Gale,[b] S. A. Goodchild,[c] D. W. Cleary,[c] S. A. Weller,[c] T. Brown[d] and P. N. Bartlett*[a]

Supplementary Information

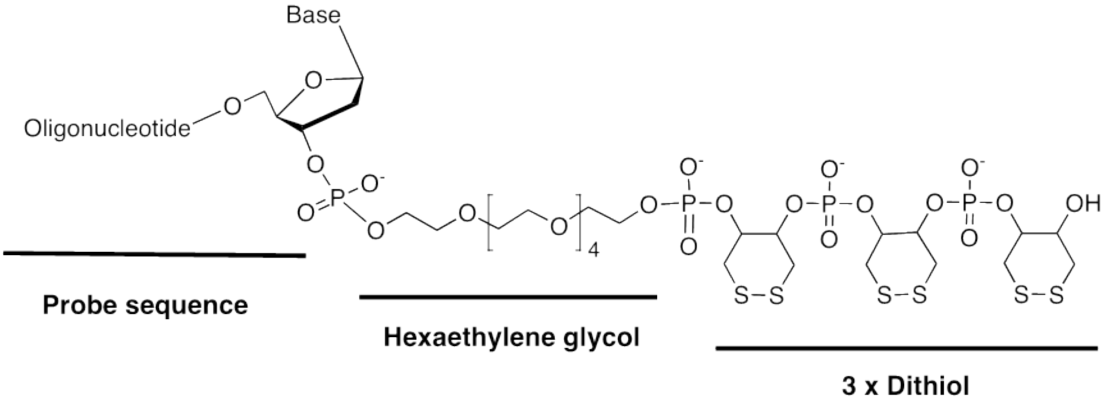

Figure S1 Structure of dithiol and hexaethylene glycol linker attached at the 3' end of the capture probes.

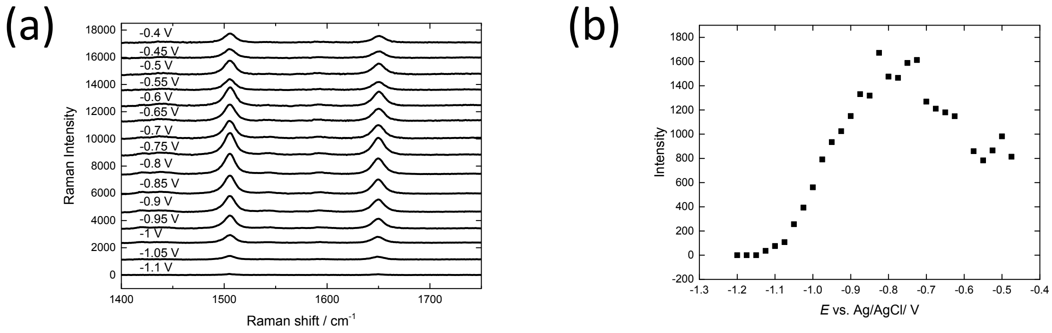

Figure S2 (a) SERS spectra of Texas Red for the PCR fragment from CO92 bacterial strain hybridised to probe-1. The spectra have been recorded at different applied potentials vs. Ag/AgCl (-0.4 V - -1.1 V). The spectra have been normalized with respect to the laser power and accumulation time. (b) Plot of the changes in the absolute signal intensities of the band at 1504 cm^{-1} as a function of applied potential. The increase in signal between -0.4 and -0.7 V is reversible and is attributed to reorientation of the Texas Red label with potential (D. K. Corrigan, N. Gale, T. Brown and P. N. Bartlett, *Angew. Chem. Int. Ed.*, 2010, **49**, 5917).

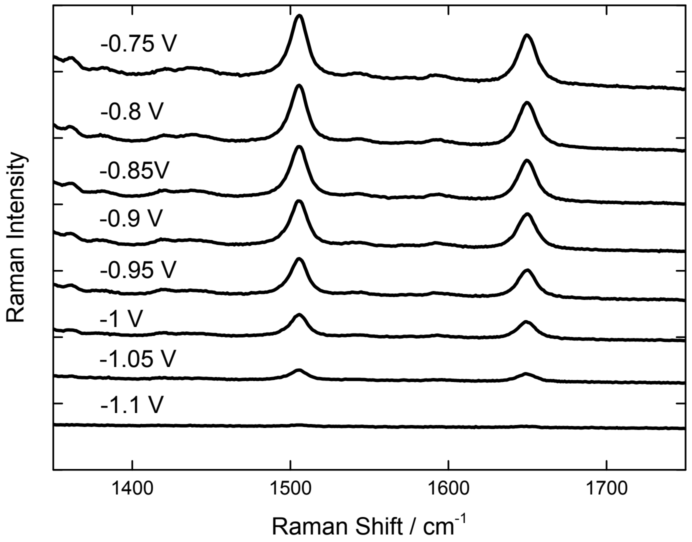

Figure S3 SER spectra of Texas Red before background subtraction for the PCR fragment from CO92 bacterial strain hybridised to probe-1. The spectra have been recorded at different applied potentials vs. Ag/AgCl as shown in the Figure.

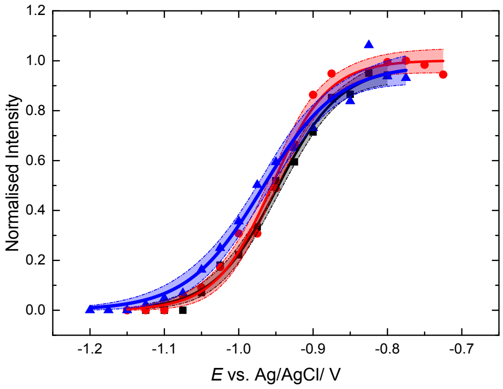

Figure S4 Electrochemical melting curves from CO92 (6 repeat units), Harbin 35 (5 repeat units), Kim (4 repeat units) hybridised to probe-1. Each set of data was fitted to equation 1. The potential was swept at a scan rate of 0.7 mV s^{-1} in 10 mM Tris buffer (pH 7.2) containing 1 M NaCl. Solid lines represent the electrochemical melting curves and dotted lines the 95% confidence limits.

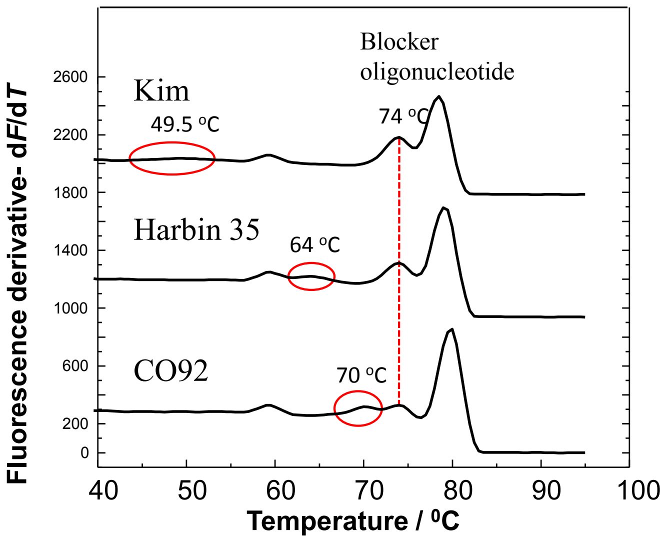

Figure S5 Fluorescence melting curves for the three strains of *Y. pestis*, CO92 (6 repeats of the VNTR sequence), Harbin (5 repeats), Kim (4 repeats) in the PCR buffer. The peaks inside the cycles show the melting temperature of the three PCR amplicons bound to probe-2 in the presence of the blocker oligonucleotide. In all cases the melting temperature of the blocker oligonucleotide is shown to be higher than that of probe-2. The melting was monitored by adding 0.6 µl of Sybr green (Biorad) to 20 µL of PCR product that contained equal amounts (0.45 µM) of probe-2 and blocker oligonucleotide.
